# Microcrystalline Cellulose-Stabilized Pickering Emulsions for Integrating Hydrophobic NADES into Agar Films: Structure–Function Relationships and Controlled Release Behavior

**DOI:** 10.3390/polym18091071

**Published:** 2026-04-29

**Authors:** Gülen Yeşilören Akal, Perihan Akbaş, Hüseyin Gençcelep

**Affiliations:** 1 Karadeniz Advanced Technology Research and Application Center, Ondokuz Mayıs University, 55200 Samsun, Türkiye; perihan.akbas@omu.edu.tr; 2 Faculty of Engineering, Department of Food Engineering, Ondokuz Mayıs University, 55200 Samsun, Türkiye; hgenccelep@omu.edu.tr

**Keywords:** agar, NADES, MCC, controlled release, biopolymer films

## Abstract

In this study, a microcrystalline cellulose (MCC)-stabilized Pickering emulsion approach was developed to integrate hydrophobic natural deep eutectic solvents (NADES; menthol:decanoic acid, 1:1 molar ratio) into agar-based biopolymer films. MCC was evaluated not only as a filler but also as a functional interfacial component governing hydrophobic phase distribution and structural organization. SEM analysis showed that MCC concentration significantly influenced morphology; films with 0.2% MCC exhibited a more homogeneous structure, whereas 0.5% MCC led to heterogeneous and irregular formations. Mechanically, films with 0.2% MCC showed higher elongation at break (16.37%) compared to 0.5% MCC (9.86%), while tensile strength remained similar (2.75–2.78 MPa). Increased MCC content enhanced surface hydrophobicity, as indicated by higher contact angle values. The 0.5% MCC films exhibited high moisture content (85%) and water solubility (93%), attributed to increased free volume and structural irregularity. Swelling index exceeded 40% in 0.2% MCC films but decreased at higher MCC levels. HS-GC-MS analysis revealed temperature-dependent controlled release of menthol, with significant release at 50 °C compared to 25 °C. Antimicrobial tests demonstrated broad-spectrum activity (8.9–24.2 mm). These results highlight MCC as an effective stabilizer for hydrophobic NADES integration and support the potential of these films for active packaging applications.

## 1. Introduction

The plastic-based food packaging industry remains one of the most robust sectors in the global food supply chain, thanks to its low cost, lightweight nature, processability, and superior barrier properties; these characteristics generate significant economic value in terms of product protection, extended shelf life, distribution efficiency, and reduced food loss. However, academic studies published over the past five years indicate that the economic impact of this sector must now be assessed not only in terms of production volume and commercial scale, but also in conjunction with the waste management burden, health risks, recycling limitations, and regulatory compliance costs that arise throughout the product lifecycle. In particular, recent reviews and systematic studies reveal that while plastics maintain their functional superiority in many packaging applications, the sustainability debate has shifted beyond material substitution to a broader framework centered on the circular economy, reuse systems, mono-material designs, life-cycle assessment, and life-cycle costing. Consequently, while the plastic-based food packaging sector remains a strong economic actor on a global scale, it has entered a transformation process where its future competitiveness will be determined not only by production capacity but also by resource efficiency, recyclability, regulatory compliance, and overall sustainability performance [1,2,3,4,5].

Growing environmental awareness has led to a rapid increase in demand for alternative packaging materials derived from biodegradable, and renewable sources because conventional plastic-based food packaging imposes an environmental burden and may have adverse effects on human health. In this context, biopolymer films based on polysaccharides, proteins, or lipids stand out because they can degrade in nature, are produced from non-toxic components, and can ensure food contact safety. However, the properties of these biopolymer-based films—such as mechanical strength, water/moisture barrier properties, gas and vapor permeability, and thermal stability—generally lag behind those of standard plastic films [6,7].

One of the major advantages of polysaccharide-based films (e.g., agar) is the ability to produce transparent, smooth-surfaced films by through casting from a simple solution. However, pure agar films have weaknesses such as brittleness, high moisture and water vapor permeability, and low thermal and mechanical stability. To address these shortcomings, agar films are reinforced with nanomaterials or enriched with hydrophobic/biologically active components to create “active/functional films” [8].

Although menthol possesses strong antimicrobial properties, it exhibits significant limitations when used directly in film systems due to its high volatility, low water solubility, and limited compatibility with hydrophilic biopolymer matrices. These characteristics lead to rapid evaporation of menthol, its non-homogeneous distribution within the film matrix, and uncontrolled release, resulting in a rapid decline in its functional efficacy. To overcome these limitations, hydrophobic natural deep eutectic solvent (NADES) systems formed by menthol with suitable hydrogen-bonding components, such as decanoic acid, have emerged as an effective alternative. NADES are systems that can transition to a liquid state at room temperature when hydrogen bond acceptor (HBA) and hydrogen bond donor (HBD) components are combined in specific ratios; they exhibit low volatility and are classified as “green solvents.” Hydrophobic menthol-fatty acid (e.g., decanoic acid)-based NADES systems are attracting attention in active packaging films because they facilitate the dissolution of lipophilic bioactive components and can exhibit strong antimicrobial effects. In such systems, menthol is stabilized through intermolecular interactions, its volatility is reduced, and it exhibits a more controlled release behavior. However, it is known that NADESs act not only as a carrier phase but also exhibit a plasticizer-like effect within the biopolymer matrix, increasing chain mobility, modifying the free volume, and influencing the physical properties of the film structure [9]. In this regard, the use of NADES offers a multifaceted approach that not only ensures the effective integration of hydrophobic antimicrobial components but also simultaneously improves the structural and functional properties of the film system. However, even though menthol-based hydrophobic NADES systems have been developed, the homogeneous and stable integration of this phase into water-based biopolymer matrices remains a significant engineering challenge. This situation highlights the necessity not only of a suitable carrier phase but also of an effective interfacial strategy to ensure the stabilization of this phase within the matrix.

Microcrystalline cellulose (MCC) is increasingly being used as a reinforcing phase in biopolymer films due to its high crystalline structure, widespread availability, relatively low cost, and biocompatibility. Recent reviews indicate that MCC can improve mechanical strength, thermal stability, and barrier properties in active food packaging systems; it can also contribute to antimicrobial functionality with appropriate formulations [10]. Furthermore, MCC is considered not only a conventional filler but also a functional component that acts as a structural regulator and influences interfacial behavior within the biopolymer matrix [11,12]. Nanocrystalline cellulose (CNC) can also be considered an alternative to MCC due to its nanoscale dimensions, high aspect ratio, and ability to form interlacing networks that enhance stress transfer within polymer matrices [13]. In parallel with these characteristics, MCC remains highly attractive, particularly in terms of availability, cost-effectiveness, and ease of processing. In this context, it contributes not only to mechanical reinforcement but also to the modulation of structural organization and interfacial interactions within biopolymer systems. Its performance is strongly dependent on dispersion quality and concentration; in particular, higher loadings may lead to particle aggregation and reduced mechanical efficiency, whereas well-dispersed MCC can effectively improve structural integrity [10].

Regarding agar–cellulose interactions, the literature indicates that MCC can induce significant structural changes in the film structure. Harnkarnsujarit and Li investigated the structure–property relationships in MCC films modified with agar and propylene glycol alginate, demonstrating that MCC significantly influences film performance. Similarly, previous studies have shown that MCC is used as an initial cellulose-based structure in agar-based composite films, and its interactions with the agar matrix are decisive for film performance. However, recent studies report that the addition of MCC does not always result in a linear improvement; while reinforcing effects are observed at low or moderate levels, performance degradation may occur at higher loadings due to particle aggregation and heterogeneous morphology. This situation demonstrates that it is not merely the “presence” of MCC that is critical, but also its distribution within the system and its interfacial behavior [14]. While reinforcing effects of MCC are generally observed at low to moderate loadings (typically ≤0.2–0.3 wt%), higher concentrations (≥0.5 wt%) have been reported to induce particle aggregation and heterogeneous morphology, ultimately leading to deterioration in mechanical and barrier performance. This indicates that not only the presence of MCC, but also its dispersion within the matrix and interfacial interactions, plays a critical role in determining overall film performance [14].

The literature indicates that phenolic compounds, nanomaterials, fatty acids, essential oils, natural solvents, and emulsion-based approaches can be used to impart water vapor and oxygen barrier properties, mechanical strength, thermal stability, and biological activity to biopolymer films [9,15]. On the other hand, emulsion systems provide a robust platform for the more stable transport of hydrophobic phases within the biopolymer matrix. It is emphasized that Pickering emulsions stabilized with biopolymer nanomaterials can simultaneously enhance antibacterial, antioxidant, barrier, and optical properties in food packaging films [16]. However, there is no comprehensive study in the literature that examines how MCC acts as an interfacial stabilizer for the integration of hydrophobic NADES into agar-based film systems and how this role relates to film morphology, mechanical performance, wettability, functionality, and controlled release.

In this study, the MCC-stabilized Pickering emulsion approach was evaluated for the integration of a menthol:decanoic acid-based hydrophobic NADES into an agar matrix, and the structural, physicochemical, and functional properties of the resulting films were systematically investigated. The primary objective of the study is to demonstrate that MCC functions not merely as a filler in this system but as a functional interfacial component that determines the distribution of the hydrophobic phase and the in-film structural organization. In this regard, the study aims to provide a more mechanistic perspective on MCC-based biopolymer film design, particularly in the context of active packaging systems carrying hydrophobic NADES.

## 2. Materials and Methods

### 2.1. Materials and Chemicals

NADES were prepared using menthol (HBA) and decanoic acid (HBD). Menthol (puriss., CAS 2216-51-5) and decanoic acid (≥98%, CAS 334-48-5) were purchased from Merck/Sigma-Aldrich (Darmstadt, Germany). Microcrystalline cellulose (MCC) (Supelco^®^, Merck, Darmstadt, Germany) with a particle size range of 5–40 µm, a density of approximately 1.5 g/cm^3^, and a pore diameter of 60 Å was used as received. Agar (food grade, Liofilchem, Roseto degli Abruzzi, Italy) was used as the film-forming biopolymer, and glycerol (%99.8, CAS 56-81-5) was used as a plasticizer. All chemicals were of analytical grade.

### 2.2. Synthesis of the Deep Eutectic Solvent

**Figure 1:**
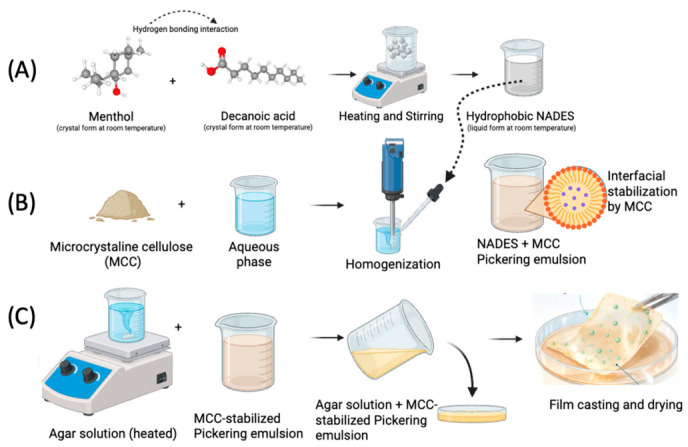
Schematic illustration of the preparation of hydrophobic NADES (**A**), followed by the formation of an MCC-stabilized Pickering emulsion (**B**), and subsequent casting and drying to obtain agar-based biopolymer films (**C**).

A hydrophobic NADES composed of menthol and decanoic acid (1:1 molar ratio) was prepared based on its previously reported hydrophobic character and ability to form stable eutectic systems (Figure 1A). The mixture was stirred at 50 °C and 180 rpm until a homogeneous liquid was obtained [17] then stored in sealed bottles in a desiccator at room temperature until use. The physicochemical and molecular characteristics of the hydrophobic NADES (menthol:decanoic acid) used in this study, including FTIR, thermal behavior, and DFT-based interaction analysis, have been comprehensively reported in our previous study [18]. Therefore, in the present work, the focus was placed on its integration into biopolymer films and its functional performance.

### 2.3. Preparation of NADES—MCC Emulsions

Microcrystalline cellulose (MCC) was selected as a particulate stabilizer due to its amphiphilic surface characteristics and its ability to adsorb at oil–water interfaces, enabling the formation of stable Pickering emulsions. NADES—MCC emulsions were prepared using MCC at concentrations of 0.2% and 0.5% (*w*/*v*) with a NADES/water ratio of 2:8 (*v*/*v*) in 10 mL systems. MCC was first dispersed in distilled water using a vortex mixer (5 min) to obtain stable suspensions (Figure 1B). The hydrophobic NADES phase (menthol–decanoic acid) was then added dropwise (1 min), followed by homogenization at 12,000 rpm for 3 min using a rotor–stator homogenizer (13 mm probe). This process yielded stable emulsions with uniformly dispersed NADES droplets.

### 2.4. Biodegradable Film Production

The agar–glycerol ratio used for film production was determined from a widely used formulation that yields a film suitable for food contact, mechanically homogeneous, and compatible with casting techniques. The agar-based film solution was prepared using 0.15 g of agar and 0.10 g of glycerol in 10 mL of distilled water [9]. Separately prepared Pickering emulsions (10 mL) were added to the film solution containing agar and glycerol on a magnetic stirrer to obtain a homogeneous mixture. Thus, the total volume of the final film formulation was fixed at 20 mL prior to film casting. During film production, the solutions were heated to 85 °C with slow stirring to ensure complete dissolution of the agar; immediately before adding the Pickering emulsions, they were cooled to 50 °C in a water bath under controlled conditions. The prepared film mixtures were poured into round glass Petri dishes (9 cm) and dried in an oven at 55 ± 5 °C for 24 h to obtain film samples (Figure 1C).

### 2.5. Structural and Physicochemical Characterization

#### 2.5.1. Electron Microscopy Imaging of Films

The microstructural properties of the film surfaces were examined using a scanning electron microscope (JSM-7001F, JEOL, Tokyo, Japan) equipped with an EDS detector (Oxford X-Max, Abingdon, UK, 80 mm^2^). The morphological features of the cross-sectional surface were examined by SEM under low-vacuum conditions. The images were obtained at an accelerating voltage of 15 kV.

#### 2.5.2. Attenuated Total Reflection Fourier Transform Infrared Spectroscopy (ATR-FTIR)

ATR-FTIR analysis was applied to determine the molecular interactions occurring in the biodegradable film. High-quality ATR-FTIR spectra were recorded using a Perkin Elmer Spectrum™ 100 FT-IR device (Waltham, MA, USA) under controlled atmospheric conditions.

### 2.6. Thermal Characterization

The thermal properties of agar films were characterized using a differential scanning calorimeter (TA Q2000 DSC, New Castle, DE, USA). For this purpose, the samples were heated to 250 °C at a heating rate of 10 °C/min and a gas flow rate of 2 mL/min under a nitrogen (N2) atmosphere, then held at 250 °C for 1 min.

### 2.7. Mechanical Properties

The tensile strength and elastic modulus of the biopolymer films were determined using a universal testing machine. Tensile strength (TS) measurements were performed according to the ASTM D882–01 standard method [19], and the analyses were conducted using an Instron tensile testing machine (Norwood, MA, USA) [20].

Tensile strength (TS) and elongation at break (EAB) were calculated according to Equations (1) and (2), respectively.

Tensile strength (MPa) = F/A(1)

Elongation at break (%) = (L − L_0_)/L_0_ (2)

Young’s modulus was determined from the slope of the initial linear portion of the stress–strain curve (Equation (3)).

Young’s modulus = σ/ε(3)

where F is the maximum force at break (N), A is the cross-sectional area of the specimen (mm^2^), L_0_ is the initial gauge length (mm), L is the length at break (mm), σ is the stress (MPa), and ε is the strain (dimensionless).

### 2.8. Surface Wettability and Water Behavior

#### 2.8.1. Determination of Water Contact Angle (WCA) 

Water contact angle (WCA) was measured using a Fytronix contact angle analyzer (Elazığ, Turkey). Film samples (30 × 30 mm) were placed on the platform, and 5 μL of distilled water was deposited onto the surface using a microsyringe. Each film sample was cut into square pieces (30 mm × 30 mm) and placed on the horizontal moving platform of a contact angle analyzer. The contact angle was recorded from both sides of the droplet.

#### 2.8.2. Determination of Moisture Content (MC), Water Solubility (WS), and Swelling Index (Si)

The moisture content (MC) of agar films was determined using the gravimetric method [8]. The initial and final masses of film samples with an area of approximately 2 cm^2^ were measured and recorded using an analytical balance. The samples were dried at 105 ± 1 °C for 24 h, and the obtained data were calculated as moisture content percentage (%) using the equation given below.

Moisture content (%) = (W_1_ − W_2_)/W1 × 100 (4)

Here, W_1_ is the initial mass, and W_2_ is the dry mass.

To determine water solubility, film samples were first dried at 105 °C for 24 h to obtain their initial dry masses (W_1_). The samples were then immersed in 50 mL of distilled water and left at room temperature for 24 h. At the end of the period, the films were removed from the medium, excess surface water was blotted off, and the films were dried again at 105 °C for 24 h to determine their final dry masses (W_2_). The solubility of the composite films in water was calculated using Equation (5) below.

Water Solubility (%) = (W_1_ − W_2_)/W1 × 100 (5)

Here, W_1_ represents the initial mass, and W_2_ represents the dry mass.

The swelling index of the films was determined by measurements taken at specific time intervals. First, film samples were cut into pieces of approximately 4.0 cm^2^ and conditioned for seven days in a desiccator containing silica gel. After conditioning, the initial weights of the samples were recorded. The samples were then immersed in 250 mL beakers containing distilled water at room temperature (25 °C) and removed from the beakers at intervals of 0.5, 1, 3, 5, 7, 10, 30, and 60 min. At each time point, the samples were dried by removing excess water from their surfaces and then weighed again. The swelling index (Si%) was calculated using Equation (6) [21].

Si (%) = (Final weight − Initial weight)/(Final weight) × 100(6)

### 2.9. Optical and UV Barrier Properties

#### 2.9.1. Transparency

To minimize variability in film thickness, all film samples were prepared by casting the same volume of solution into Petri dishes of equal diameter. The film thickness was measured with a digital micrometer at five randomly selected points on each film, avoiding the edges. It was determined that the thickness differences between films produced under the same casting conditions were within the measurement uncertainty limits; therefore, the thickness data obtained for all films were evaluated together and presented as a single average value.

To determine the transparency of the films, samples were cut to 4 mm × 10 mm, placed in a cuvette, and their light transmittance was measured at a wavelength of 600 nm using a UV spectrophotometer. Transparency values were calculated using the relevant equation.

Transparency = logT/b(7)

Here, T is the light transmittance at 600 nm and b is the film thickness (mm).

#### 2.9.2. Ultraviolet-Visible Absorbance and Transmittance

The UV–visible absorbance and transmittance properties of the films were determined using a UV–visible spectrophotometer (Shimadzu UV-1800, Kyoto, Japan). Film samples were cut into 4 mm × 10 mm pieces, placed in a spectrophotometer cell, and their optical properties were measured over the wavelength range 300–850 nm.

### 2.10. Determination of Volatile Compound Release (HS-GC–MS)

The release behavior of volatile components from agar-based films containing NADES was evaluated using headspace gas chromatography–mass spectrometry (HS-GC–MS). Prior to analysis, film samples were cut into small pieces and placed directly into headspace vials, which were then sealed airtight. Headspace analyses were performed without an internal standard, and the results were evaluated comparatively and semi-quantitatively based on peak intensities. HS-GC–MS analyses were performed using a Shimadzu GC-2010 gas chromatograph coupled to a GC-MS-QP2010 Ultra mass spectrometer (Kyoto, Japan). During the separation process, the column oven temperature was initially set to 40 °C and maintained at this temperature for 2 min. The temperature was then raised to 240 °C at a rate of 4 °C/min and held at this temperature for 10 min. The injector temperature was set to 250 °C, and injections were performed in split mode (split ratio 15:1). Helium was used as the carrier gas, and the column flow rate, linear velocity, and column pressure were set to 1.44 mL/min, 43.4 cm/s, and 80 kPa, respectively. The total flow rate was set at 26.0 mL/min, and the purge flow rate was set at 3.0 mL/min. The mass detector was operated in electron ionization (EI) mode; the ion source and interface temperatures were set at 200 °C and 250 °C, respectively. Data were collected in scan mode over the 40–550 *m*/*z* range at a scan speed of 1111 *m*/*z* s^−1^. The solvent cut time was set to 2.5 min, and data were collected between 3.0 and 62.0 min. The detector gain was set to 0.98 kV in relative mode. Headspace analyses were performed at two different temperatures (25 °C and 50 °C) to investigate the temperature-dependent behavior of volatile compound release. The obtained chromatograms were compared using retention times and peak intensities; thus, volatile compound release profiles were qualitatively and semi-quantitatively evaluated across different film formulations. This approach allows evaluation of the temperature-dependent release behavior of volatile compounds, providing insight into the controlled release functionality of the films.

### 2.11. Biological Activity

#### 2.11.1. Determination of Antimicrobial Activity

The disk diffusion method was used to determine antimicrobial activity. *Bacillus cereus* (NTCC 7464), *Bacillus subtilis* (ATCC 6633), *Enterococcus faecalis* (ATCC 29212), and *Staphylococcus aureus* (ATCC 25923) were inoculated onto Mueller–Hinton agar. The Gram-negative bacteria *Klebsiella pneumoniae* (ATCC 700603), *Pseudomonas aeruginosa* (ATCC 9027), *Yersinia enterocolitica* (ATCC 23717), *Acinetobacter baumannii* (ATCC 19606), *Escherichia coli* (ATCC 25922) and the yeast *Candida albicans* were used. Amikacin 30 µg was used as a positive control. Bacterial and yeast suspensions were adjusted to 10^8^ colony-forming units (CFU/mL), and 100 μL of each suspension was added to Petri dishes. Films cut into 6-mm-diameter circles were transferred onto the agar. After incubation at 37 °C for 24 h, the inhibition diameters formed around the films were measured.

#### 2.11.2. Determination of Antifungal Activity

Antifungal activity was evaluated using the agar disk diffusion method. An *Aspergillus niger* strain was used as the test organism. The fungal culture was incubated at 25 °C for 21 days on Sabouraud Dextrose Agar (SDA). The spore suspension was prepared in sterile 0.85% NaCl solution and adjusted to a density of 0.5 McFarland standard (1 × 10^6^ spores/mL). The prepared suspension was spread homogeneously on the SDA surface. Sterile 10-mm-diameter films were placed on the agar surface, and inoculated Petri plates were used as negative controls. The plates were incubated at 30 °C, and the diameters of the inhibition zones formed around the films were measured in millimeters (mm) using a digital caliper [22].

### 2.12. Statistical Analysis

The antimicrobial activity and physico-mechanical properties of the films were evaluated in triplicate using independently prepared samples. Statistical differences among the mean values were analyzed by one-way analysis of variance (ANOVA), followed by Duncan’s multiple range test at a significance level of *p* < 0.05 using SPSS software (version 20.0). Graphical representations of the data were generated using OriginPro 2025 software.

## 3. Results

### 3.1. Film Formation, Structural Integrity, and Molecular Interactions

#### 3.1.1. SEM Analyses

**Figure 2:**
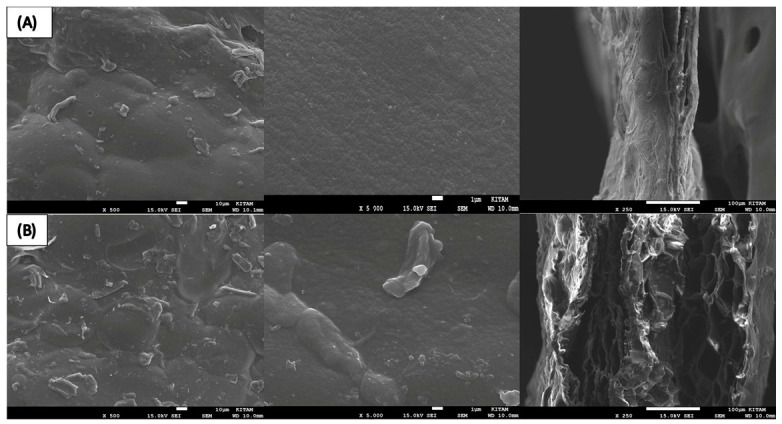
Surface (×500 and ×5000) and cross-section (×250) SEM images of agar-based films containing different contents of MCC. (**A**) 0.2% MCC, (**B**) 0.5% MCC.

In this study, the morphological properties of agar-based films containing hydrophobic NADES were systematically evaluated by scanning electron microscopy (SEM) as a function of MCC concentration. SEM images of the film surface (×500 and ×5000) and cross-section (×250) reveal that MCC plays a decisive role in film formation, influencing internal structural organization, compactness, and thickness distribution (Figure 2). SEM images (surface at ×500 and ×5000 magnifications, and cross-sections at ×250) reveal notable differences in film morphology depending on MCC content. High-magnification (×5000) SEM surface images revealed that the 0.2% MCC formulation (A) exhibited a relatively smoother and more homogeneous surface, indicating a more uniform distribution of MCC within the agar matrix. In contrast, the film with 0.5% MCC (B) showed a rougher surface with visible aggregates, suggesting partial particle agglomeration at higher concentrations. This observation suggests a more homogeneous dispersion of MCC within the agar matrix at lower loading levels.

Cross-sectional images further support this observation; the 0.2% MCC film displayed a more compact and continuous internal structure, whereas the 0.5% MCC film exhibited a more heterogeneous and less organized morphology. The SEM observations suggest that MCC contributes not only as a structural filler but may also influence the dispersion and spatial distribution of the hydrophobic phase within the agar matrix, indicating a potential role in interfacial stabilization. No visible cracking or structural discontinuities were observed in any of the film samples, indicating that the incorporation of MCC-stabilized NADES did not compromise the structural integrity of the agar matrix.

#### 3.1.2. Attenuated Total Reflection–Fourier Transform Infrared Spectroscopy (ATR-FTIR)

**Figure 3:**
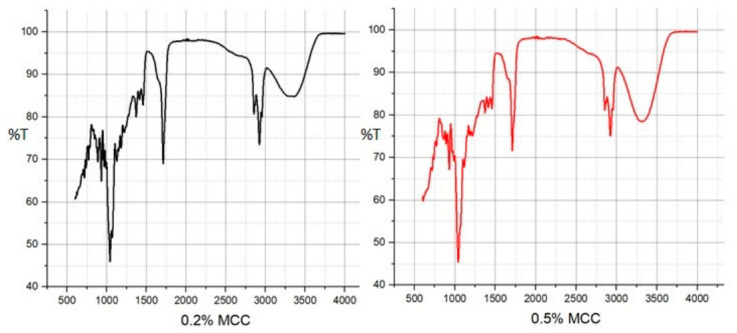
Representative ATR-FTIR spectra of agar-based films containing different contents of MCC.

Fourier transform infrared (FTIR) spectroscopy was employed to investigate the possible interactions between agar, MCC, and hydrophobic NADES components within the film matrix. The broad band observed in the 3200–3400 cm^−1^ region in all samples corresponds to the characteristic stretching vibrations of hydroxyl (–OH) groups in agar and microcrystalline cellulose (MCC) (Figure 3). Variations in the width and intensity of this band may indicate changes in intermolecular interactions among the film components, which could be associated with hydrogen bonding [23]. The band observed at 2920–2950 cm^−1^ is attributed to C–H stretching vibrations of aliphatic structures, such as menthol and decanoic acid, supporting the presence of hydrophobic NADES components within the film. Although weak C–H signals may also arise from the polysaccharide matrix, the increased intensity in this region upon NADES incorporation suggests a contribution from the hydrophobic phase.

A band observed at 1710–1711 cm^−1^ can be assigned to the C=O stretching vibration of carboxylic acid groups. Similar bands around 1700 cm^−1^ have been reported in menthol–fatty acid-based NADES systems, and are generally associated with the presence of hydrogen-bonded acid structures [24]. However, this observation should be interpreted as supportive rather than definitive evidence of NADES formation.

The region between 1040–1070 cm^−1^ corresponds to C–O–C (glycosidic linkage) and C–O stretching vibrations typical of polysaccharides such as agar and cellulose [23,24,25] confirming the structural integrity of the biopolymer matrix.

### 3.2. Mechanical Performance and Structural Strength

**Figure 4:**
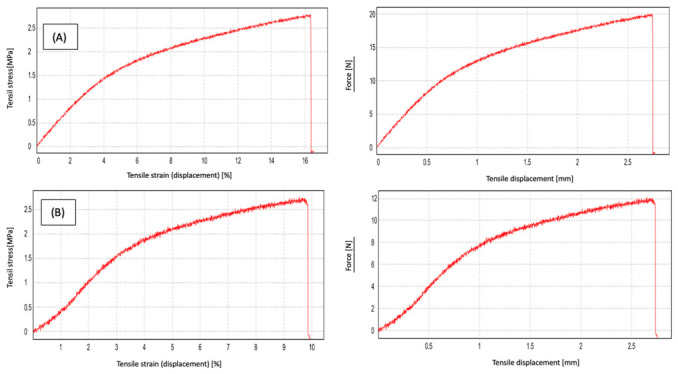
Tensile stress–strain (**left**) and force–displacement (**right**) curves of films (**A**) 0.2% MCC, and (**B**) 0.5% MCC.

Tensile test results indicate that MCC concentration has a decisive effect on the mechanical behavior of agar/NADES-based films (Figure 4). Tensile strength (TS), elongation at break (EAB), and Young’s modulus represent, respectively, the maximum load the film can bear, its deformation capacity prior to fracture, and its stiffness; the combined evaluation of these parameters is critical for a comprehensive understanding of the mechanical performance of film systems.

According to the results, the film sample containing 0.2% MCC exhibited a higher elongation at break (EAB: 16.37%), whereas increasing the MCC content to 0.5% resulted in a significant decrease in elongation at break (EAB: 9.86%). In contrast, no significant increase was observed in tensile strength (TS) and Young’s modulus values. This indicates that the addition of MCC does not linearly improve mechanical performance beyond a certain concentration but may instead introduce structural heterogeneity (Table 1).

Although tensile strength values did not show a significant change with increasing MCC concentration, distinct differences were observed in elongation at break; this indicates a transition from a more flexible structure to a stiffer one. This suggests that MCC primarily affects the structural organization and deformation behavior of the films rather than their ultimate strength. Furthermore, it should be noted that the sole purpose of biodegradable film systems is not to achieve the mechanical strength of traditional petroleum-based plastics, but rather to provide functional performance such as biodegradability and active ingredient delivery.

These findings suggest that the relationship between MCC concentration and film performance is not strictly linear; therefore, future studies may focus on a broader concentration range to better elucidate the balance between dispersion, aggregation, and mechanical behavior.

### 3.3. Thermal Properties

**Figure 5:**
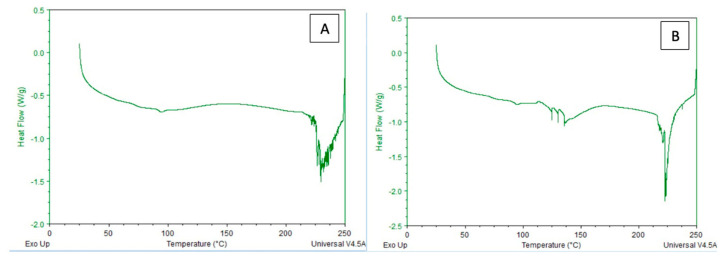
DSC thermograms of agar-based films (**A**) 0.2% MCC, and (**B**) 0.5% MCC.

DSC analyses indicate that the thermal behavior of agar-based films containing hydrophobic NADES changes significantly depending on the microcrystalline cellulose (MCC) concentration (Figure 5). The broad endothermic peak observed in the 30–80 °C range in all samples is associated with the removal of weakly bound or free water, as commonly reported in agar- and cellulose-based biopolymer films. The literature indicates that the endothermic transitions observed in this temperature range represent the desorption of water bound to hydrophilic functional groups [26,27,28]. The smaller endothermic transitions observed in the 90–110 °C range can be attributed to hydrogen bond rearrangements occurring between the agar chains and the NADES components. This indicates that MCC plays a role not only as a structural filler but also as a component that influences molecular interactions within the agar–NADES system.

Notably, at high MCC concentrations, the appearance of multiple endothermic events in the DSC curves and the peaks exhibiting a sharper and more dispersed profile are striking. This phenomenon can be explained by the heterogeneous distribution of micron-sized MCC particles in the matrix, the formation of local stress fields, and the establishment of limited interfacial interactions with the agar–NADES network. Similarly, in the literature, it has been reported that thermal transitions in polysaccharide-based film systems using MCC or larger-sized cellulose derivatives are more heterogeneous and spread over a wider temperature range [27].

### 3.4. Surface Wettability and Moisture Behavior

#### 3.4.1. Water Contact Angle (WCA) Analysis

**Figure 6:**
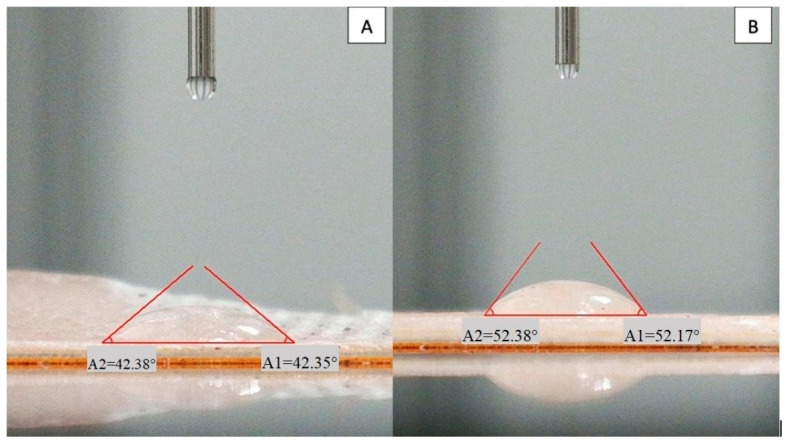
Representative static water contact angle (WCA) images of agar-based films (**A**) 0.2% MCC, and (**B**) 0.5% MCC.

The surface wettability of films is a critical parameter for both moisture management and the controlled release of active ingredients from the food-contact surface. The results indicate that the microcrystalline cellulose (MCC) concentration significantly modulates the hydrophilic–hydrophobic balance of the agar/NADES film system (Figure 6).

The findings indicate that surface properties can be tailored to specific targets by adjusting the MCC concentration. While relatively more hydrophilic surfaces are obtained at lower MCC concentrations, more hydrophobic surfaces can be created with higher MCC content. This provides a significant advantage, particularly in food packaging applications, in terms of controlling moisture transfer, surface stability, and optimizing the release kinetics of active ingredients.

The numerical WCA values with standard deviations are presented in Table 2. As shown in the table, increasing the MCC content from 0.2% to 0.5% resulted in a significant increase in the contact angle (from 42.3° to 52.2°), indicating a transition toward a more hydrophobic surface. The statistical difference between the samples (*p* < 0.05) confirms that MCC concentration plays a key role in modulating surface wettability.

#### 3.4.2. Moisture Content and Water Solubility

**Figure 7:**
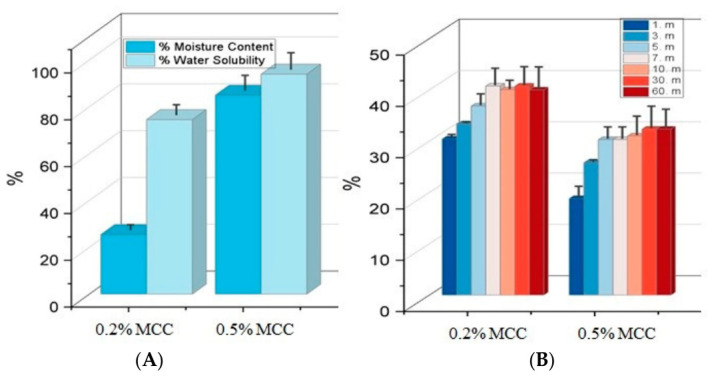
Physicochemical characterization of agar/NADES-based films reinforced with MCC: (**A**) Moisture content (MC%) and water solubility (WS%) values, (**B**) Swelling index (Si) kinetics over 60 min. The labels (m) represent extraction time in minutes.

The moisture content and water solubility of biopolymer films are fundamental physicochemical properties that determine the barrier performance, mechanical strength, and environmental degradability of packaging materials. As shown in Figure 7A, changes in MCC concentration significantly affected the agar–NADES matrix’s interaction with water. An increase in moisture content and water solubility values was observed as the MCC ratio rose from 0.2% to 0.5%. The fact that 0.5% MCC exhibited a moisture content of 85% and a water solubility of 93% indicates that the increased MCC content enhances the film structure’s interaction with water.

This is thought to be related to the fact that a high MCC loading creates a more disordered structure in the matrix, increasing the free volume regions and facilitating the retention of water molecules within the film structure. Similarly, it has been reported in the literature that an increase in water content in biopolymer film structures affects polymer chain reorganization and leads to structural relaxation.

#### 3.4.3. Swelling Index (Si)

The swelling index of films is an important parameter in terms of indicating the resistance of the biopolymer network structure to water molecules and the dimensional stability of the packaging material in humid environments. As shown in Figure 7B, all film formulations exhibited rapid water absorption within the first 7 min and subsequently reached a steady state. Among the samples containing MCC, the sample with 0.2% MCC exhibited higher swelling values across all time intervals, while the sample with 0.5% MCC showed relatively lower swelling rates.

The fact that 0.2% MCC reached swelling values exceeding 40% particularly from the 7th minute onward indicates that the structure formed at a low MCC concentration is more susceptible to water penetration. In contrast, the decrease in swelling rate in 0.5% MCC, obtained by increasing the MCC ratio, suggests that a higher filler concentration creates physical constraints between polymer chains, thereby limiting the matrix’s water absorption capacity.

### 3.5. Transparency and UV Barrier Performance

The optical properties and UV protection capacity of films are critical parameters in food packaging design, as they enable the consumer to see the product and protect light-sensitive components. In this study, transparency was expressed using the transparency index (A_600_/b), an absorbance-based parameter; a decrease in the index value indicates increased optical transmittance. When the transparency index and UV–visible absorption spectra are evaluated together, it was observed that the MCC concentration plays a decisive role in the optical performance of the agar/NADES matrix.

**Figure 8:**
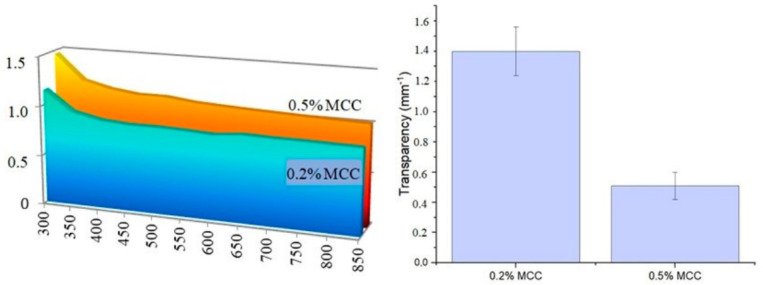
Optical characterization of agar/NADES-based films reinforced with MCC: UV-visible absorbance spectra between 300–850 nm, and Transparency indices at 600 nm.

In the transparency index graph (Figure 8), considering that lower values represent higher light transmittance, the sample containing 0.5% MCC exhibited the lowest index value (0.51 mm^−1^ band) and was thus identified as the most transparent structure among the MCC-containing formulations. This low refractive index obtained in the sample containing 0.5% MCC indicates an increase in light transmittance within the film structure. When evaluated in conjunction with SEM cross-sectional images (Figure 2) and the low tensile strength value (2.75 MPa), this finding can be attributed to the combined effects of film thickness, internal structural heterogeneity, and light–matrix interactions. It has been reported in the literature that structural homogeneity and particle dispersion in biopolymer films have a direct effect on optical transmittance [29].

According to UV–Vis spectroscopic analysis, upon examining the UV absorption spectrum, it is observed that samples containing MCC exhibit a certain level of UV absorption in the 300–400 nm range. In particular, the fact that the sample containing 0.5% MCC maintains its UV absorbance despite providing high transparency in the visible region indicates that this formulation can offer a balanced combination of visible light transmittance and UV barrier properties.

### 3.6. Evaluation of Menthol Release Behavior

**Figure 9:**
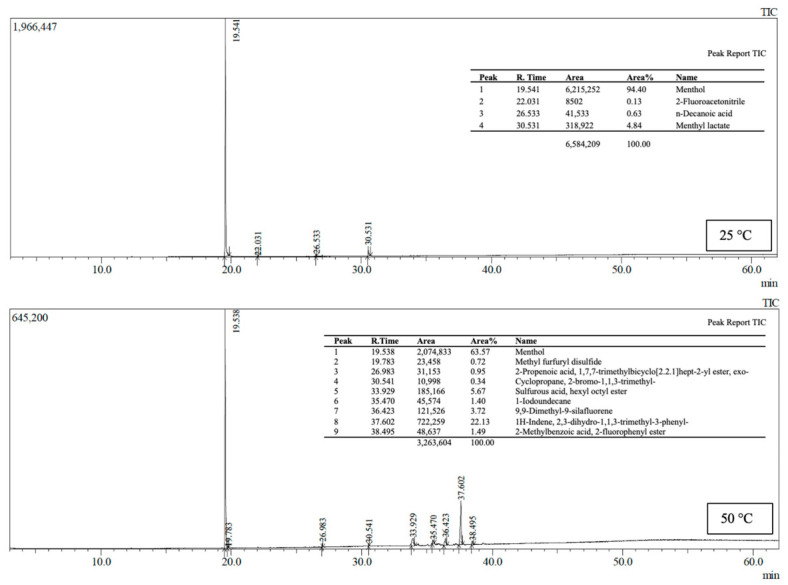
HS-GC–MS total ion chromatogram (TIC) and corresponding peak report of volatile compounds detected in film 0.2% MCC at 25 °C and 50 °C.

HS-GC–MS analyses revealed that menthol release in MCC-containing agar/NADES film systems is not solely temperature-dependent but is also strongly controlled by the formulation-specific matrix structure. In the 0.2% MCC formulation (Figure 9), menthol accounted for 94.40% of the total signal at 25 °C, establishing it as the dominant volatile component, while components such as menthyl lactate (4.84%) and n-decanoic acid (0.63%) remained at low levels. However, when the temperature was raised to 50 °C, the relative proportion of menthol dropped to 63.57%, and concurrently, the number and concentration of secondary volatile components increased significantly. This indicates that thermal effects in the formulation not only increase menthol release but also facilitate the release of other volatile components previously confined within the matrix into the headspace [30,31].

**Figure 10:**
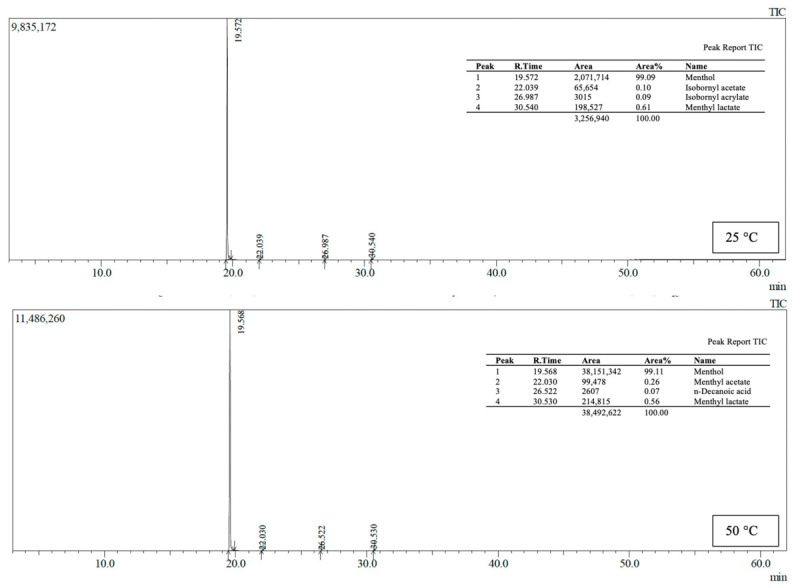
HS-GC–MS total ion chromatogram (TIC) and corresponding peak report of volatile compounds detected in film 0.5% MCC at 25 °C and 50 °C.

In contrast, the 0.5% MCC formulation exhibited a distinctly more selective and menthol-dominant release profile (Figure 10). At 25 °C and 50 °C, menthol accounted for nearly the entire volatile profile at 99.09% and 99.11%, respectively, and the menthol peak area increased from approximately 3.21 × 10^7^ to 3.82 × 10^7^. Nevertheless, the proportion of secondary components remained below 1% at both temperatures. This result indicates that, in this formulation, menthol release increases with rising temperature, while the release of other volatile components is largely suppressed. This suggests that the MCC–agar/NADES matrix structure can retain components other than menthol more effectively and that a selective diffusion mechanism is activated under thermal stimulation.

### 3.7. Evaluation of Biological Activity

#### 3.7.1. Antimicrobial Activity

**Figure 11:**
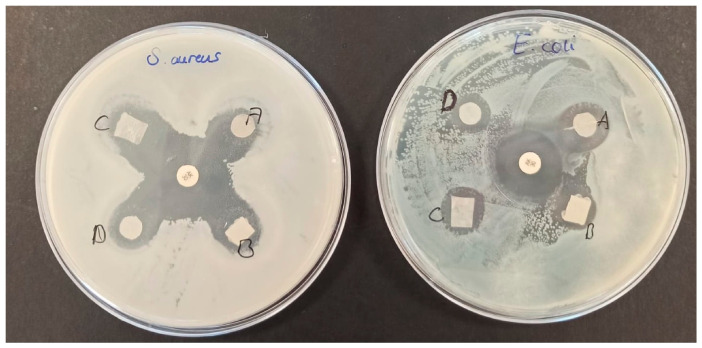
Images of inhibition zones demonstrating antibacterial activity against *E. coli* and *S. aureus*. A and B represent parallel replicates of the 0.2% MCC formulation, C and D represent parallel replicates of the 0.5% MCC formulation.

When all bacterial strains were evaluated together, the inhibition zone diameters ranged from 8.9 to 24.2 mm (Figure 11). Among the MCC-containing film formulations, the highest antibacterial activity was observed in the 0.2% MCC formulation, which provided significant inhibition particularly against *Yersinia enterocolitica* (24.2 mm) and *Acinetobacter baumannii* (20.8 mm). These results demonstrate that while films containing MCC exhibit broad-spectrum antibacterial activity, the efficacy varies depending on the type of microorganism.

**Figure 12:**
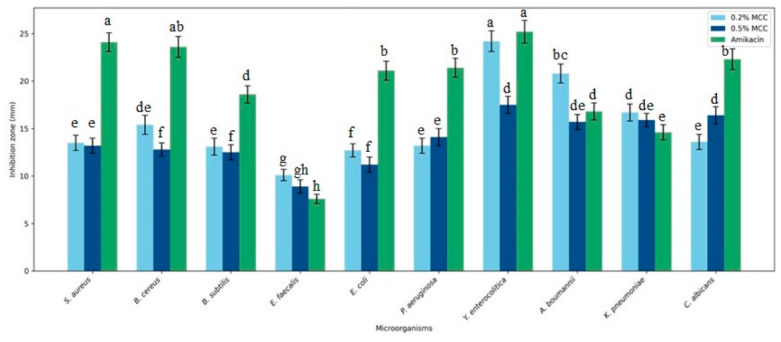
Antibacterial activity of agar/NADES-based films reinforced with microcrystalline cellulose (MCC) against selected Gram-positive and Gram-negative microorganisms. Different letters (a–h) above the bars indicate statistically significant differences between treatments for each microorganism (*p* < 0.05), based on one-way ANOVA followed by Tukey’s post hoc test.

Figure 11 shows distinct inhibition zones against *E. coli* and *S. aureus*, confirming the antibacterial efficacy of the MCC-containing films tested. Quantitative analyses revealed that the inhibition zone diameters ranged from approximately 10.5–21.1 mm for *E. coli* and 11.4–24.1 mm for *S. aureus* (Figure 12).

When formulations containing MCC were compared with one another, 0.2% MCC exhibited broader-spectrum activity and a higher inhibitory effect, particularly against Gram-negative pathogens. In contrast, 0.5% MCC demonstrated relatively lower antibacterial activity. This suggests that the increased MCC concentration may have reduced the release of antimicrobial components by creating denser or more irregular regions in the film structure that limit the diffusion of the active ingredient.

#### 3.7.2. Antifungal Activity

Antifungal activity was evaluated using the agar disk diffusion method over a 21-day incubation period, with *Aspergillus niger* used as the test organism. The approximate inhibition zone diameters measured on the first day were 10 mm for Film A and 8 mm for Film B. Initially, Film A exhibited higher antifungal activity, while the lowest inhibition was observed in Film B. The comparative antifungal evaluation of the films is presented in Table 3.

By the end of the third day, the diameter of the inhibition zone around all films had decreased to approximately 1 mm. However, spore formation was observed around both films. It is believed that the decrease in zone diameter is due to a drop in concentration resulting from the antifungal active ingredient diffusing into the medium over time.

**Figure 13:**
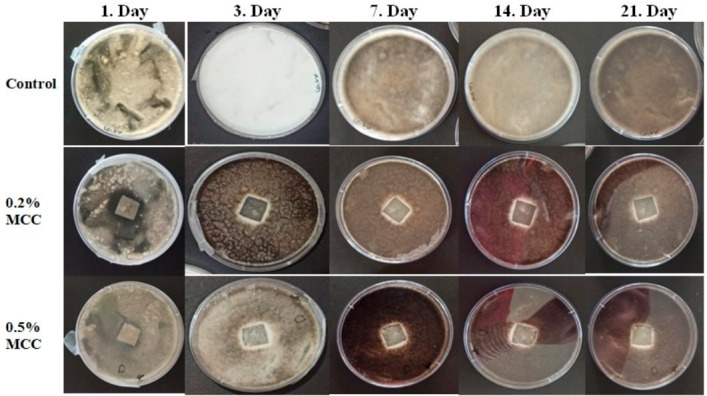
Daily photographs of the antifungal activity test conducted with *A. niger* fungus.

*A. niger* is a filamentous fungus that grows rapidly and produces dense spores, making it a robust model microorganism for evaluating antifungal performance. Nevertheless, images obtained on days 7, 14, and 21 showed that the mycelial density had not fully advanced toward the center of the film. The fact that the films were not completely covered at the end of the 21-day incubation period indicates that the film formulations containing MCC exhibit long-term antifungal activity. This can be explained by the active ingredient creating continuous fungistatic suppression due to its controlled and slow release (Figure 13).

## 4. Discussion

SEM cross-sectional analysis revealed that increasing MCC content led to a more heterogeneous and irregular internal morphology, characterized by the disruption of continuous pore structures. This observation indicates that higher MCC loadings alter matrix organization, likely due to particle aggregation and uneven distribution within the polymer network, consistent with previous reports on cellulose-reinforced biopolymer systems [26]. FTIR analysis confirmed the successful incorporation of the NADES phase into the agar matrix. The characteristic C=O stretching band observed at 1710–1711 cm^−1^ corresponds to the carboxylic acid groups of the menthol–decanoic acid system and serves as a key indicator of DES formation. Additionally, the 1040–1070 cm^−1^ region reflects the polysaccharide backbone (C–O–C and C–O vibrations), confirming the structural integrity of the agar–cellulose matrix [23,24,25]. Mechanical analysis showed that MCC concentration plays a dual role in determining film performance. The 0.2% MCC film exhibited a more ductile behavior with higher elongation, whereas the 0.5% MCC film displayed a brittle response with reduced de-formation capacity. This transition can be attributed to increased particle aggregation and stress concentration at higher filler loadings, which limits effective stress transfer within the matrix [11,23]. Thermal analysis further demonstrated that MCC acts as a structural regulator within the agar–NADES system. The observed degradation onset in the 200–250 °C range aligns with typical agar-based films, while variations in thermal behavior suggest that particle distribution and interfacial interactions significantly influence thermal transitions [32]. Surface wettability results indicated that increasing MCC content from 0.2% to 0.5% increased the contact angle, reflecting a shift toward a more hydrophobic surface. This behavior is associated with increased surface roughness and topographical heterogeneity induced by micron-sized MCC particles, which reduce the effective contact area between water droplets and the film surface [26]. Despite this increase in surface hydrophobicity, higher MCC content led to increased moisture content (75–85%) and water solubility (82–93%), while the swelling index decreased. This apparent contradiction can be explained by distinguishing between surface and bulk properties. While surface wettability decreases, the internal structure becomes more heterogeneous and contains greater free-volume regions, facilitating water retention and matrix disintegration. At the same time, the more rigid and particulate structure restricts polymer chain mobility, limiting swelling behavior. Therefore, these parameters are complementary rather than contradictory, collectively describing water–film interactions at different structural levels [9]. On the other hand, the coarser and more heterogeneous structure formed by MCC in the matrix, consistent with the particulate morphology observed in SEM analyses, may have created micro-pores that could facilitate the diffusion of water molecules between agar chains. It has been reported in the literature that microcrystalline cellulose can affect water transport by creating porosity and free volume in biopolymer matrices [33]. However, at higher MCC loadings, the increase in particle density and the resulting higher degree of packing in the matrix may have partially restricted the water penetration path. Optical analysis showed that MCC content influences light transmission through changes in internal structure. While the particulate nature of MCC can induce light scattering, a more compact particle distribution at higher concentrations may enhance visible light transmittance. At the same time, MCC-containing films maintained UV absorption in the 300–400 nm range, indicating a balance between transparency and UV protection, which is advantageous for food packaging applications [26]. It has been reported in the literature that the amount and distribution homogeneity of the filler in cellulose-based biopolymer films significantly affect their optical properties [26,34].

Release behavior analysis demonstrated that MCC content plays a critical role in controlling the release profile of menthol. Depending on the formulation, the system can provide either broader-spectrum release or more selective, controlled release behavior. This highlights the tunable nature of MCC-based Pickering systems, where both release rate and selectivity can be adjusted [25]. Based on antimicrobial activity analysis, notable inhibition zones were also obtained against Gram-positive bacteria. Although Gram-positive bacteria lack an outer membrane despite having a thick peptidoglycan layer, this facilitates the diffusion of active components into the cell, a phenomenon similarly reported in many active packaging studies [35]. Antimicrobial results confirmed that the films exhibit broad-spectrum activity against both Gram-positive and Gram-negative bacteria. High inhibition zones, particularly against *Y. enterocolitica*, suggest that the active components may act by disrupting membrane permeability or compromising cell integrity. This mechanism is consistent with previous reports on active packaging systems involving nanoparticles or bioactive compounds [36].

Overall, the results demonstrate that MCC functions not only as a reinforcing agent but also as a key structural and interfacial component that governs morphology, water interaction, mechanical behavior, and active compound release within the agar–NADES film system.

## 5. Conclusions

This study presents an innovative approach for the effective integration of NADES containing hydrophobic components with high antimicrobial activity into water-based biopolymer film systems. In particular, the solubility and phase separation issues encountered when directly integrating a component with strong antimicrobial properties, such as menthol, into the agar matrix were successfully overcome using a Pickering emulsion system stabilized with microcrystalline cellulose (MCC). This approach enables the stable and functional transport of hydrophobic active ingredients within biopolymer films.

The results obtained demonstrate that MCC is not merely a structural filler but also functions as a critical interfacial regulator that governs the distribution and retention of the hydrophobic NADES phase within the matrix. This structure allows menthol to be retained in a controlled manner within the film matrix and released via a mechanism triggered by an increase in temperature. HS-GC–MS results indicate that the release behavior varies depending on the formulation; while broader-spectrum volatile release was observed in some systems, others yielded a menthol-dominant release profile with high selectivity. This demonstrates that the developed film system serves not only as a carrier for the active ingredient but also as a platform capable of controlling the release kinetics and efficacy of this component.

This controlled release mechanism emerges as the key factor directly enhancing the antimicrobial performance of the developed films. The findings indicate that agar/NADES film systems containing MCC exhibit broad-spectrum antibacterial activity against both Gram-positive and Gram-negative bacteria and also possess antifungal activity. The controlled retention of menthol within the matrix prevents rapid release of the active ingredient, ensuring long-term biological activity and thereby enhancing the system’s functional efficiency.

This study demonstrates that integrating menthol-based hydrophobic NADES into biopolymer film systems improves antimicrobial efficacy and film properties. The Pickering emulsion approach stabilized with MCC offers a robust and flexible platform that not only incorporates hydrophobic components into the matrix but also enables their controlled and targeted release. In this regard, the developed system has strong potential for active food packaging applications and makes a significant contribution to both scientific originality and industrial applicability.

In addition to these findings, future research should focus on further optimizing the MCC-stabilized Pickering emulsion system to enhance the scalability and industrial applicability of the developed films. In particular, systematic investigations on the long-term stability of the films under real food storage conditions, as well as migration and safety assessments of NADES components, are essential for practical implementation. Moreover, exploring different types of hydrophobic NADES formulations and bioactive compounds could expand the functional versatility of the system. Advanced characterization of release kinetics under dynamic environmental conditions (e.g., varying humidity and temperature) would provide deeper insight into the controlled release mechanisms. In particular, future studies may address barrier properties (e.g., vapor and gas permeability), biodegradability, migration behavior. Finally, integrating this system into real food packaging trials will be a critical step toward validating its performance and commercial potential.

## Figures and Tables

**Table 1:** Tensile strength (TS), elongation at break (EAB), and Young’s modulus of agar-based films containing MCC.

Sample Code	TS (MPa)	EAB (%)	Young’s Modulus (MPa)
A (0.2% MCC)	2.78 ± 0.03 ^a^	16.37	41.86 ± 0.27 ^a^
B (0.5% MCC)	2.75 ± 0.07 ^a^	9.86	39.64 ± 0.35 ^ab^

Tensile strength (TS), elongation at break (EAB), and Young’s modulus were determined from stress–strain curves. Different letters within the same column indicate statistically significant differences (*n* = 3, *p* < 0.05).

**Table 2:** Water contact angle (WCA) values of films.

Sample	WCA (°)
0.2% MCC	42.3 ± 1.8 ^b^
0.5% MCC	52.2 ± 2.1 ^a^

Different superscript letters (a, b) within the same column indicate statistically significant differences (*p* < 0.05).

**Table 3:** Comparative Antifungal Evaluation of Films.

Film	Initial Zone	Spore Formation	21-Day Effect	Overall Evaluation
A (0.2% MCC)	Moderate (10 mm)	Present	Limited growth	Medium
B (0.5% MCC)	Low (8 mm)	Present	Limited growth	Weakest start

## Data Availability

The original contributions presented in the study are included in the article, further inquiries can be directed to the corresponding author.

## Abbreviations

The following abbreviations are used in this manuscript:

ATR-FTIR	Attenuated Total Reflection–Fourier Transform Infrared Spectroscopy
CFU	Colony-Forming Units
DES	Deep Eutectic Solvent
DSC	Differential Scanning Calorimetry
EAB	Elongation at Break
EI	Electron Ionization
GC–MS	Gas Chromatography–Mass Spectrometry
HBA	Hydrogen Bond Acceptor
HBD	Hydrogen Bond Donor
HS-GC–MS	Headspace Gas Chromatography–Mass Spectrometry
MCC	Microcrystalline Cellulose
MC	Moisture Content
NADES	Natural Deep Eutectic Solvent
ROS	Reactive Oxygen Species
SEM	Scanning Electron Microscopy
Si	Swelling Index
TS	Tensile Strength
UV–Vis	Ultraviolet–Visible Spectroscopy
WCA	Water Contact Angle
WS	Water Solubility

## References

[1] Ada E., Kazancoglu Y., Gozacan-Chase N., Altin O. (2023). Challenges for circular food packaging: Circular resources utilization. Appl. Food Res..

[2] Dolci G., Puricelli S., Cecere G., Tua C., Fava F., Rigamonti L., Grosso M. (2024). How does plastic compare with alternative materials in the packaging sector? A systematic review of LCA studies. Waste Manag. Res..

[3] Bokor B. (2025). Legal analysis of the EU regulatory framework on circular economy and sustainability principles in plastic food packaging. Clean. Waste Syst..

[4] Haslinger A.-S., Nhu T.T., Cadena E., Thomassen G., Dewulf J., Huysveld S. (2026). Life cycle assessment and life cycle costing of emerging circular flexible plastic food and non-food packaging. Resour. Conserv. Recycl..

[5] Ghasemlou M., Barrow C.J., Adhikari B. (2024). The future of bioplastics in food packaging: An industrial perspective. Food Packag. Shelf Life.

[6] Mostafavi F.S., Zaeim D. (2020). Agar-based edible films for food packaging applications—A review. Int. J. Biol. Macromol..

[7] Periyasamy T., Asrafali S.P., Lee J. (2025). Recent Advances in Functional Biopolymer Films with Antimicrobial and Antioxidant Properties for Enhanced Food Packaging. Polymers.

[8] Kanmani P., Rhim J.-W. (2014). Antimicrobial and physical-mechanical properties of agar-based films incorporated with grapefruit seed extract. Carbohydr. Polym..

[9] Akal G.Y., Akbaş P. (2025). Functional biodegradable agar based films from polyphenol-rich matcha extracts via deep eutectic solvents: From microstructure to bioactivity. Int. J. Biol. Macromol..

[10] Narayanaperumal S., Suyambulingam I., Divakaran D., Senthamaraikannan P., Selvan A., Avudaiappan S., Ayrilmis N., Eyupoglu S. (2026). Recent advances in microcrystalline cellulose-reinforced polymer composites: Extraction, properties, applications, and sustainability perspectives. Int. J. Biol. Macromol..

[11] Bangar S.P., Esua O.J., Nickhil C., Whiteside W.S. (2023). Microcrystalline cellulose for active food packaging applications: A review. Food Packag. Shelf Life.

[12] Muhammed A.P., Thangarasu S., Oh T.H. (2023). Green interconnected network structure of chitosan-microcrystalline cellulose-lignin biopolymer film for active packaging applications. Int. J. Biol. Macromol..

[13] Marcuello C., Foulon L., Chabbert B., Molinari M., Aguié-Béghin V. (2018). Langmuir–Blodgett Procedure to Precisely Control the Coverage of Functionalized AFM Cantilevers for SMFS Measurements: Application with Cellulose Nanocrystals. Langmuir.

[14] Harnkarnsujarit N., Li Y. (2017). Structure–property modification of microcrystalline cellulose film using agar and propylene glycol alginate. J. Appl. Polym. Sci..

[15] Ezati P., Khan A., Priyadarshi R., Bhattacharya T., Tammina S.K., Rhim J.-W. (2023). Biopolymer-based UV protection functional films for food packaging. Food Hydrocoll..

[16] Ding F., Long S., Huang X., Shi J., Povey M., Zou X. (2024). Emerging Pickering emulsion films for bio-based food packaging applications. Food Packag. Shelf Life.

[17] Ribeiro B.D., Florindo C., Iff L.C., Coelho M.A., Marrucho I.M. (2015). Menthol-based eutectic mixtures: Hydrophobic low viscosity solvents. ACS Sustain. Chem. Eng..

[18] Yeşilören Akal G. (2026). Menthol-based hydrophobic deep eutectic solvents: Design, chemical interaction, and performance evaluation for microplastic extraction from salt matrices. Int. J. Environ. Anal. Chem..

[19] (2017). Standard Test Method for Tensile Properties of Thin Plastic Sheeting.

[20] Chaubey A., Aadil K.R., Jha H. (2021). Synthesis and characterization of lignin-poly lactic acid film as active food packaging material. Mater. Technol..

[21] de Barros Vinhal G.L.R.R., Silva-Pereira M.C., Teixeira J.A., Barcia M.T., Pertuzatti P.B., Stefani R. (2021). Gelatine/PVA copolymer film incorporated with quercetin as a prototype to active antioxidant packaging. J. Food Sci. Technol..

[22] Bansod S., Rai M. (2008). Antifungal activity of essential oils from Indian medicinal plants against human pathogenic Aspergillus fumigatus and A. niger. World J. Med. Sci..

[23] Wang X., Guo C., Hao W., Ullah N., Chen L., Li Z., Feng X. (2018). Development and characterization of agar-based edible films reinforced with nano-bacterial cellulose. Int. J. Biol. Macromol..

[24] Sekkal M., Legrand P., Huvenne J.P., Verdus M.C. (1993). The use of FTIR microspectrometry as a new tool for the identification in situ of polygalactanes in red seaweeds. J. Mol. Struct..

[25] Elgharbawy A.A.M., Syed Putra S.S., Khan H.W., Azmi N.A.N., Sani M.S.A., IIah N.A., Hayyan A., Jewaratnam J., Basirun W.J. (2023). Menthol and Fatty Acid-Based Hydrophobic Deep Eutectic Solvents as Media for Enzyme Activation. Processes.

[26] Atef M., Rezaei M., Behrooz R. (2014). Preparation and characterization agar-based nanocomposite film reinforced by nanocrystalline cellulose. Int. J. Biol. Macromol..

[27] Shankar S., Rhim J.-W. (2016). Preparation of nanocellulose from micro-crystalline cellulose: The effect on the performance and properties of agar-based composite films. Carbohydr. Polym..

[28] Zhang J., Lei W., Chen J., Liu D., Tang B., Li J., Wang X. (2018). Enhancing the thermal and mechanical properties of polyvinyl alcohol (PVA) with boron nitride nanosheets and cellulose nanocrystals. Polymer.

[29] Felfel R.M., Hossain K.M.Z., Kabir S.F., Liew S.Y., Ahmed I., Grant D.M. (2018). Flexible and transparent films produced from cellulose nanowhisker reinforced agarose. Carbohydr. Polym..

[30] Casalini S., Montanari F., Giacinti Baschetti M. (2023). Diffusion of Thyme, Cinnamon and Oregano essential oils in different nanocellulose matrices. Carbohydr. Polym. Technol. Appl..

[31] Frydrysiak E., Śmigielski K., Kunicka-Styczyńska A., Frydrysiak M. (2024). Investigation of Releasing Chamomile Essential Oil from Inserts with Cellulose Agar and Microcrystalline Cellulose Agar Films Used in Biotextronics Systems for Lower Urinary Tract Inflammation Treatment. Materials.

[32] Kassab Z., Abdellaoui Y., Salim M.H., Bouhfid R., Qaiss A.E.K., El Achaby M. (2020). Micro- and nano-celluloses derived from hemp stalks and their effect as polymer reinforcing materials. Carbohydr. Polym..

[33] Thirugnanasambandham I., Roychowdhury P., Rao R.P., Janakiraman A.K., Dhanasekaran M., Singh S.K., Kuppusamy G. (2026). Role and Applications of Biodegradable Nanocomposites vs. Nondegradable Nanocomposites in Biomedical Applications. Nanocomposites and Nanomaterials in Biomedical Applications.

[34] Oun A.A., Rhim J.-W. (2015). Effect of post-treatments and concentration of cotton linter cellulose nanocrystals on the properties of agar-based nanocomposite films. Carbohydr. Polym..

[35] Gyawali R., Ibrahim S.A. (2014). Natural products as antimicrobial agents. Food Control.

[36] Akbaş P., Erdoğan H. (2025). Production and characterization of agar based biodegradable food films developed from deep eutectic pea hull (*Pisum sativum*) extract. Int. J. Biol. Macromol..

